# Increasing Age, the Existence of Comorbidities, and Corticosteroid Treatment in Combination With Antiviral Therapy Prolongs the Recovery of SARS-COV-2-Infected Patients, Measured as the Conversion From Positive to Negative rtPCR: A 239 Patients' Retrospective Study

**DOI:** 10.3389/fmed.2020.575439

**Published:** 2020-11-12

**Authors:** Sheng Zhu, Yaxiong Huang, Wei Tang, Andreas K. Nussler, Fang Zheng

**Affiliations:** ^1^Department of Trauma and Reconstructive Surgery, BG Trauma Center Tuebingen, Siegfried Weller Institute for Trauma Research, Eberhard Karls University Tuebingen, Tuebingen, Germany; ^2^The First Hospital of Changsha, Changsha, China

**Keywords:** COVID19, SARS-CoV-2, RTPCR, age, comorbidity, corticosteroid

## Abstract

**Background:** Severe acute respiratory syndrome (COVID-19), caused by severe acute respiratory syndrome coronavirus 2 (SARS-COV-2), has become a global pandemic in the past months. An overall defined treatment has not yet been established. Therefore, it is important to summarize and report treatment experiences and identify patient groups that have a significantly higher risk of an adverse clinical outcome.

**Methods:** Two hundred thirty-nine COVID-19 patients were recruited from January 25 to February 15, 2020. Demographic, clinical, laboratory, treatment management, and outcome data obtained from patients' medical records were evaluated.

**Results:** Patients who recovered from PCR positive to negative within 2 weeks had significantly lower erythrocyte sedimentation rate (ESR) and higher C-reactive protein (CRP) levels than those recovered post 2 weeks. During antiviral treatment, COVID-19 patients with older age, comorbidities, and corticosteroid treatment required a significantly longer time to turn from PCR positive to negative COVID-19 result.

**Conclusion:** PCR tests are of great importance to evaluate the recovery of COVID-19-positive patients, and ESR could be an indirect indicator to monitor SARS-COV-2 activity. Furthermore, our data suggest that older age, the existence of comorbidities, and corticosteroid treatment of COVID-19 patients during antiviral treatment could prolong the duration of conversion from SARS-COV-2 positive to negative.

## Introduction

In December 2019, severe acute respiratory syndrome coronavirus 2 (SARS-COV-2) was isolated, and the resultant disease was termed as a novel coronavirus severe acute respiratory syndrome (COVID-19) (1). In the meantime, COVID-19 has become a global pandemic, causing enormous damage to human health and the economy worldwide. One hundred eighty-eight countries/regions are affected, and more than 30 million people have been confirmed positive for COVID-19 (WHO COVID-19 Weekly Epidemiological Update, 5 Oct, 2020). Currently, many therapeutic agents for COVID-19 have not proven to be effective or are still in clinical trials as confirmed cases continue to increase worldwide (2). It is promising that China, severely affected by SARS-COV-2, has shown a static or declining trend since the pandemic began. By September 2020, a large number of COVID-19-positive patients had recovered in China and other countries. However, recent data indicated that a second COVID-19 wave is ongoing in Europe (3) and most likely in other parts of the world (4). It is therefore essential to summarize and report the treatment experiences for salvaged patients for each affected area and to carry out further studies.

Due to the similarity of the clinical features between SARS-CoV-2 infection and other viral diseases (5, 6), merely confirming COVID-19 patients based on clinical symptoms does not seem to be sufficient. It is believed that the majority of patients experiences asymptomatic or mild courses of the infection (7). Under these circumstances, real-time polymerase chain reaction (rtPCR) screening tests have become the gold standard for both confirming COVID-19-positive patients and evaluating the clinical outcome as well as discharging patients (8, 9). It is now generally accepted that SARS-CoV-2-positive patients can be discharged from the hospital if two consecutive rtPCR results are negative at intervals of 24 h (10). The time between positive and negative results of rtPCR determines the hospital stay and the associated costs for society. Given the considerable shortage of medical and financial resources worldwide (11), it will become increasingly important to identify patient groups that have a significant risk of extended hospital stays and to choose the right treatment strategy.

The Hunan province bordered directly on the Hubei province and was one of the first provinces in China where SARS-COV-2 infections occurred. To date, 1,014 out of 1,019 confirmed SARS-COV-2 patients have recovered in Hunan province (12). In this study, we report the clinical experience in epidemiology, clinical features, and treatment management of 239 recovered COVID-19 patients from Changsha First Hospital. One focus of this work reports on the clinical course between SARS-COV-2-positive and SARS-COV-2-negative rtPCR results.

## Methods

### Patients

In this retrospective, single-center study, we recruited patients from January 25 to February 15, 2020, at The First Hospital of Changsha in Changsha, Hunan province, China, which is a designated hospital for COVID-19. Inclusion criteria of a nucleic acid PCR test for patients in this study were the following: (1) epidemiological conditions—(i) history of travel to or residence in Wuhan district or potential contact with people from Wuhan within 14 days, (ii) contact with confirmed Covid-19 patients within 14 days, (iii) contact with people from Wuhan district or pandemic area within 14 days, (iv) contact with people who had a fever or other respiratory symptoms within 14 days, and (v) clustered cases (two or more confirmed cases in small areas like families, schools, offices or workplace within 14 days); (2) clinical condition—(i) patients with fever or respiratory symptoms, (ii) radiological changes in the lung, and (iii) white blood cells decreased or stayed normal, while lymphocyte counts decreased. In addition, patients with at least one epidemiological condition and one clinical condition, as well as patients with no epidemiological conditions but with two clinical conditions, were tested by rtPCR. Inclusion criteria for patient's evaluation of the present study were as follows: (i) patients who were diagnosed with COVID-19 according to a positive rtPCR result at the time of admission; (ii) symptoms recovered after hospitalization; (iii) rtPCR was negative twice at intervals of 24 h before discharge; and (iv) received antiviral drugs treatments. The study was approved by The First Hospital of Changsha Hospital Ethics Committee, and written informed consent was obtained from all patients before enrolment.

### Procedure

rtPCR was routinely done for patients on days 1, 3, 7, and 10 after admission and tested every 3 days if patients stayed more than 10 days after admission. The clinical classification of patients was based on the Diagnosis and Treatment Protocol for Novel Coronavirus Pneumonia in China (Trial Version 7) (13). Patients with mild symptoms and no sign of pneumonia on imaging were considered as mild cases, patients with fever and respiratory symptoms and radiological findings of pneumonia were considered as moderate cases, while patients meeting any of the following criteria were considered as severe cases: (1) respiratory distress (≧30 breaths/min); (2) oxygen saturation ≤ 93% at rest; (3) arterial partial pressure of oxygen (PaO2)/fraction of inspired oxygen (FiO2) ≦300 mmHg (l mmHg = 0.133 kPa); and (4) patients with chest imaging that showed obvious lesion progression within 24–48 h (>50%). Demographic, clinical, laboratory parameters, treatment management, and outcome data were obtained from patients' medical records.

### Treatment Management

All patients received supportive and antiviral treatments. Supportive treatments were staying in bed, protein–calorie supplementation, maintenance of hydroelectrolyte balance, and oxygen inhalation were routinely given to all COVID-19-positive patients. For severe patients, oxygen therapy, mechanical ventilation, rescue therapy, circulation support, replacement therapy, or immunotherapy was used. Antiviral drugs were applied according to the following schema: Lopinavir/Ritonavir (200/50 mg per pill, twice a day) and Arbidol (200 mg, three times a day) were used for no longer than 10 days; α-interferon (five million *U* or equivalent dose each time for adults, twice a day) and Novaferon (20 μg, twice a day) were applied by aerosol inhalation; three or more drugs combination should be avoided (13). All patients received at least one antiviral drug during hospitalization, and the drug could be changed if rtPCR results were positive for more than 1–2 weeks. Patients with type 1 respiratory failure, increasing lung lesion, and severe symptoms (shock, organ dysfunction) received additionally methylprednisolon, 40 mg, twice a day.

### Outcomes

The number of COVID-19 patients in our study was summarized by age, gender, and symptoms (Table 1). The time from the rtPCR positive to the negative result was defined as the time between the admission date with SARS-COV-2 positive and the first time a negative rtPCR result during hospitalization. The time of an rtPCR result from positive to negative was compared among groups divided by demographic data (age, gender), medical history (with or without chronic diseases), symptoms (with or without typical symptoms), blood inflammation markers [erythrocyte sedimentation rate (ESR) and C-reactive protein (CRP)], and treatment management (antiviral drugs and corticosteroids).

**Table 1:** Demographics of 239 patients admitted to The First Hospital (January 25–February 25) with COVID-19.

**GENDER [NUMBER OF PATIENTS (PERCENTAGE)]**	
Female	121 (50.6%)
Male	118 (49.4%)
Age [years (range)]
Mean	44.15 (1–84)
Mean in female	46.94 (1–84)
Mean in male	44.08 (8–82)
**Age [NUMBER OF PATIENTS (PERCENTAGE)]**	
≤18	11 (4.6%)
18–29	28 (11.7%)
30–39	46 (19.2%)
40–49	54 (22.6%)
50–59	41 (17.2%)
60–69	38 (15.9%)
≥70	21 (8.8%)
**CLASSIFICATION [NUMBER OF PATIENTS (PERCENTAGE)]**	
Non-severe patients	201 (84.1%)
Severe patients	38 (15.9%)

### Statistical Analysis

Results are represented in the tables and figures (mean ± SEM). The Mann–Whitney *U* test was used to compare two single groups. The difference among multiple groups was compared by the Kruskal–Wallis H test, followed by Dunn's multiple comparison test. Statistical analysis was performed using the GraphPad Prism Software (Version 8, El Camino Real, CA, USA). *p* < 0.05 was considered as the minimum level of statistical significance.

## Results

### Demographics

All patients (239 in total) with SARS-COV-2 infection confirmed by rtPCR were included in this study. The average age is 44.15, and middle-aged patients accounted for the main proportion. There were no gender differences in the selected study group (Table 1).

### The Duration for rtPCR Result From Positive to Negative

All patients were tested by rtPCR and confirmed SARS-COV-2 positive at the time of hospital admission, and reviewed on days 1, 3, and 7 and every 3 days afterwards when necessary. Most patients (72.8%) in our study cohort recovered from a positive COVID-19 rtPCR result to a negative one within 1–4 weeks after hospital admission (Table 2).

**Table 2:** The duration from nucleic acid PCR positive to negative.

**Mean time (days)**	**16**
≤1 week	35 (14.6%)
1–2 weeks	97 (40.6%)
2–4 weeks	77 (32.2%)
>4 weeks	30 (12.6%)

### Patients' Symptoms Did Not Affect the Duration of rtPCR Result From Positive to Negative

**Figure 1:**
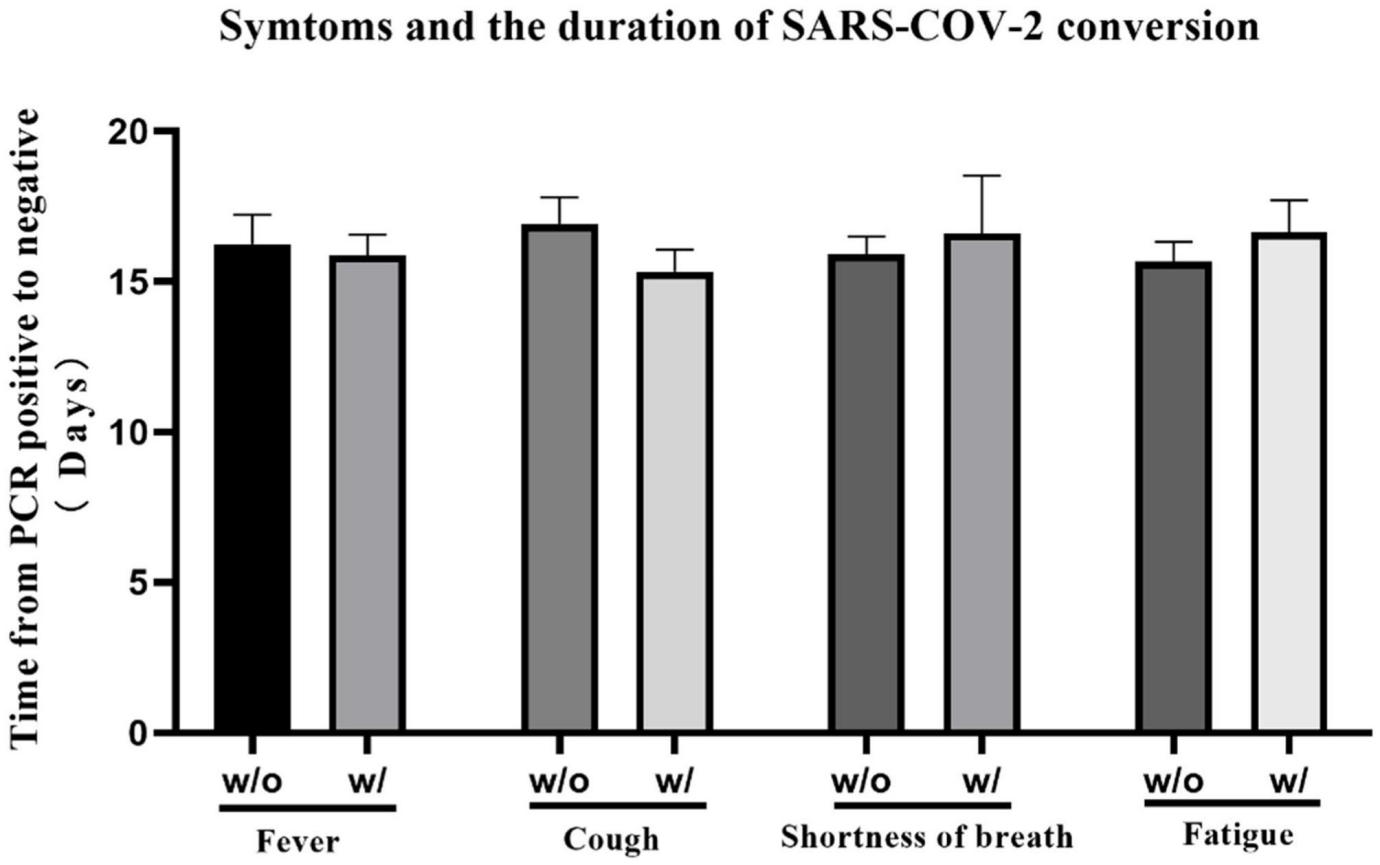
The time for real-time PCR (rtPCR) result transition from positive to negative was compared between patients with or without specific symptom at the onset. The average time of patients with/without fever was 16.24 ± 0.985/15.88 ± 0.696, *p* = 0.545; the average time of patients with/without cough was 16.92 ± 0.882/15.33 ± 0.738, *p* = 0.064; the average time of patients with/without shortness of breath was 15.91 ± 0.589/16.60 ± 1.925, *p* = 0.682; the average time of patients with/without fatigue was 15.67 ± 0.659/16.63 ± 1.074, *p* = 0.770. No significant differences were observed among the different study groups.

We summarized patients' distribution of each symptom at onset respectively, and the overview of multiple symptoms. Fever (67.3%), cough (58.2%), fatigue (33.9%), and shortness of breath (12.6%) were the most common symptoms, and most patients (60.3%) experienced more than one symptom at the onset. Rhinorrhea seems not related to COVID-19 (Table 3). We also compared the time for rtPCR result from positive to negative between patients with and without four of the most common symptoms (fever, cough, fatigue, and shortness of breath). As depicted in Figure 1, there was a significant difference in the clinical symptoms and the time of change for an rtPCR result from positive to negative (Figure 1).

**Table 3:** Clinical symptoms of patients at admission.

**TYPICAL SYMPTOMS [NUMBER OF PATIENTS (PERCENTAGE)]**	
Fever	161 (67.3%)
Cough	139 (58.2%)
Fatigue	81 (33.9%)
Shortness of breath	30 (12.6%)
Pharyngalgia	27 (11.3%)
Myalgia	23 (9.6%)
Diarrhea	20 (8.4%)
Headache	18 (7.5%)
Dizziness	10 (4.2%)
Nausea and vomiting	8 (3.3%)
Rhinorrhea	5 (2.1%)
Without symptoms	17 (7.1%)
With only 1 symptom	65 (17.2%)
With 2 symptoms	70 (23.9%)
With more than 2 symptoms	87 (36.4%)

### Patients With Lung Lesion Progression Needed More Time to Recover From a Positive rtPCR Result to a Negative One During Hospitalization

**Figure 2:**
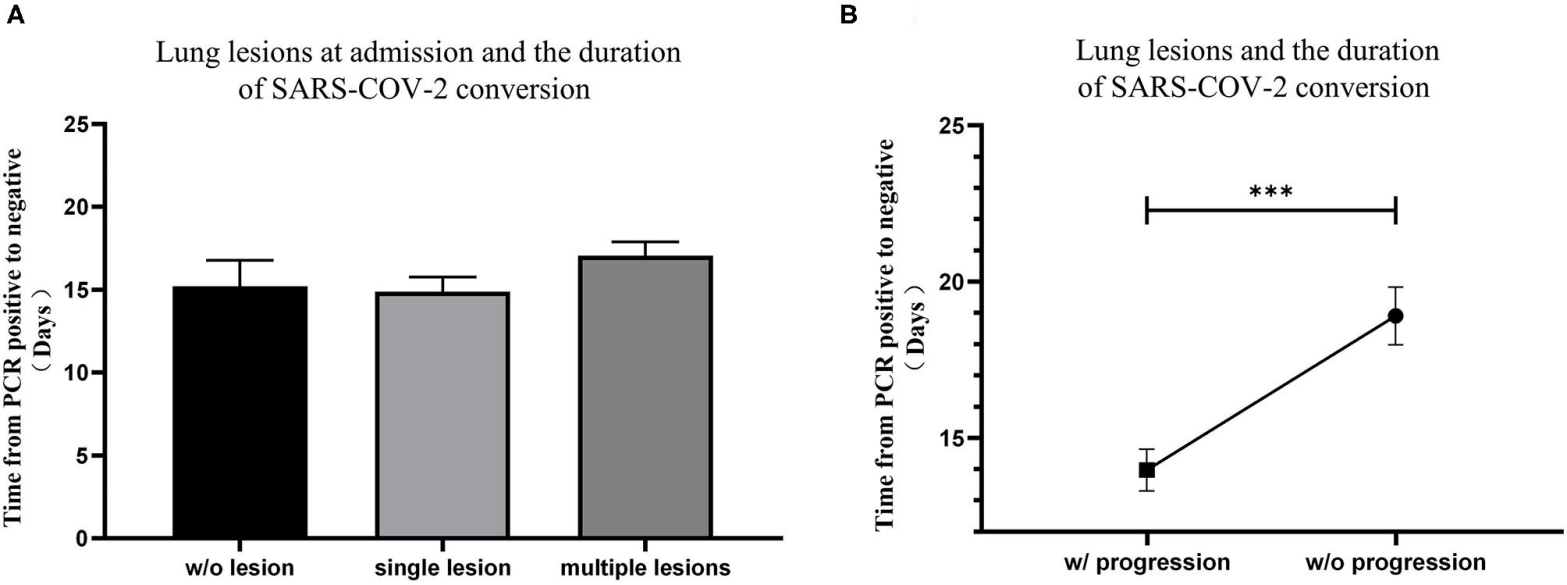
The relationship between lung lesions measured by CT and the conversion time from real-time PCR (rtPCR) positive to negative. **(A)** The conversion time from rtPCR positive to negative of patients with no lung lesion, single lung lesion, and multiple lung lesions was compared. **(B)** The conversion time from rtPCR positive to negative of patients with and without progression of lung lesions was compared. Data are represented the mean ± SEM. The significance was determined as ***p < 0.001.

Chest computed tomography (CT) scans were routinely performed on patients in the study to determine lung lesions at the time of hospital admission and reviewed every 3–5 days afterwards. At the time of hospital admission, 24 patients (10.0%) had no lung lesions, 96 patients (40.2%) had single lung lesion, and 119 patients (49.8%) had multiple lung lesions. No significant difference was found among lung lesions of patients at admission (Figure 2A). Although all patients' lung lesions improved significantly at discharge in our study, 98 patients (41.0%) experienced progression of their lung lesions during hospitalization. It is worth noting that patients who experienced lung lesion progression took significantly more time to convert rtPCR results (Figure 2B).

### Blood Inflammation Makers Could Be Useful Indicators for the Duration of Change for rtPCR From Positive to Negative

**Figure 3:**
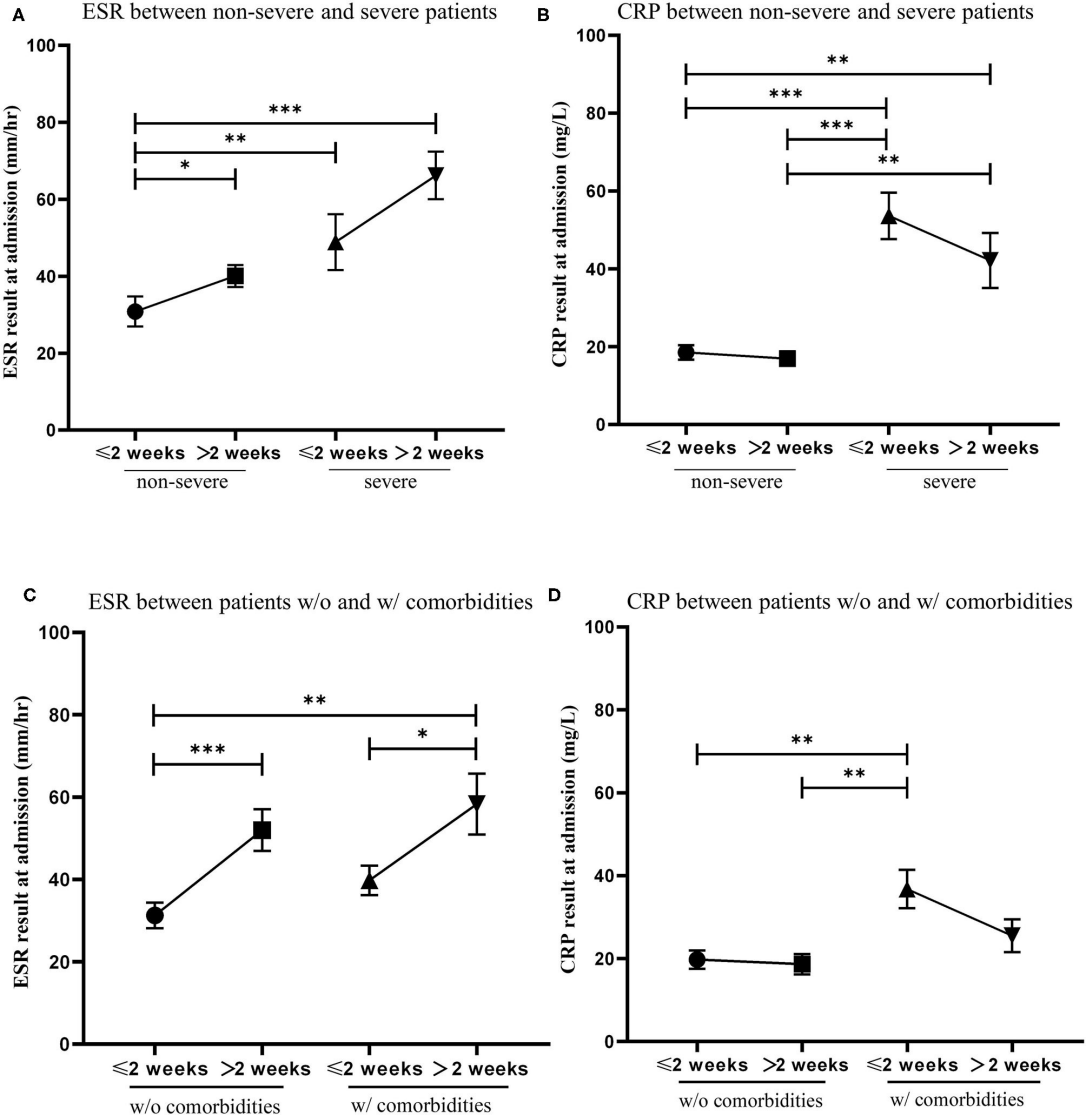
The relationship of the level of erythrocyte sedimentation rate (ESR) and C-reactive protein (CRP) at admission and the conversion time from real-time PCR (rtPCR) positive to negative. **(A)** Patients were divided into two groups by non-severe and severe cases. Blood test results of ESR was compared between patients recovered from rtPCR positive to negative within 2 weeks and those recovered over 2 weeks. **(B)** Patients were divided into two groups by non-severe and severe cases. Blood test results of CRP was compared between patients recovered from rtPCR positive to negative within 2 weeks and those recovered over 2 weeks. **(C)** Patients were divided into two groups by the presence of comorbidities. Blood test results of ESR was compared between patients recovered from rtPCR positive to negative within 2 weeks and those recovered over 2 weeks. **(D)** Patients were divided into two groups by the presence of comorbidities. Blood test results of CRP was compared between patients recovered from rtPCR positive to negative within 2 weeks and those recovered over 2 weeks. Data are represented the mean ± SEM. The significance was determined as *p < 0.05, **p < 0.01, and ***p < 0.001.

ESR and CRP are classical test parameters that indirectly measures the degree of inflammation in the blood (14). We compared ESR and CRP levels at admission of patients to turn a positive rtPCR result to negative within 2 weeks with patients that needed more than 2 weeks to turn a positive rtPCR result to negative. Patients were first divided into non-severe and severe cases. We found that severe patients had significantly higher levels of ESR and CRP than non-severe patients. Patients who recovered from a positive rtPCR result to negative within 2 weeks had lower ESR levels and higher CRP levels than those who recovered over 2 weeks (Figures 3A,B). Patients were then divided by the presence of comorbidities. We found that patients who recovered from a positive rtPCR result to negative within 2 weeks had significantly lower ESR levels than those who recovered over 2 weeks in both groups. Moreover, we found that patients with comorbidities had higher CRP levels than patients without comorbidities, and patients who recovered from a positive rtPCR result to negative within 2 weeks had slightly higher CPR levels than those who recovered over 2 weeks in both groups (Figures 3C,D).

### COVID-19 Patients With Age Above 50, Comorbidities, and Corticosteroid Treatment Needed Longer Time to Recover From a Positive rtPCR Result to a Negative One Under Antiviral Drug Treatments

**Figure 4:**
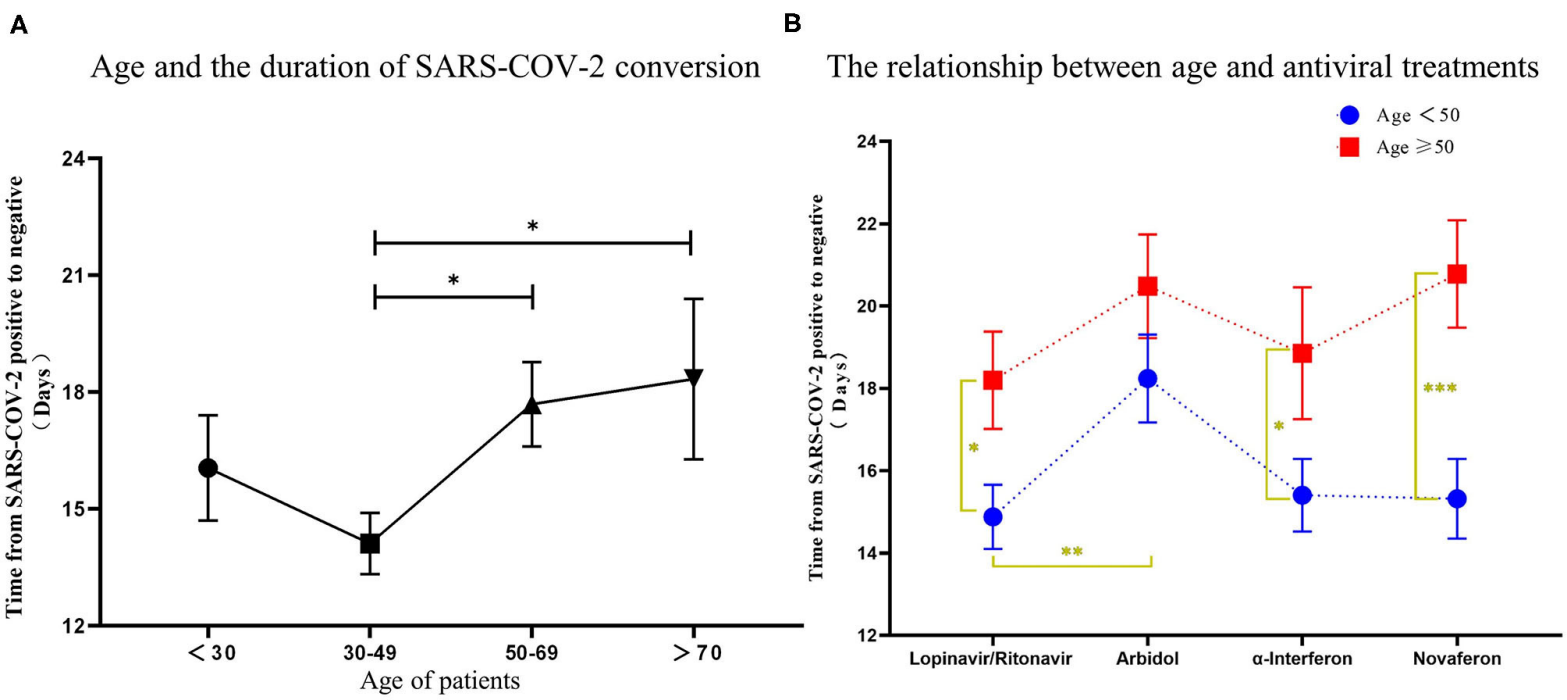
The relationship between age and the conversion time from real-time PCR (rtPCR) positive to negative during antiviral treatment. **(A)** Patients were grouped by age <30 years, 30–50 years, 50–70 years, and over 70 years, and the conversion time from rtPCR positive to negative was compared. **(B)** Patients were divided by different antiviral drugs used, and the time was compared for rtPCR positive to negative results in patients under and over the age of 50. Data are represented the mean ± SEM. The significance was determined as *p < 0.05, **p < 0.01, and ***p < 0.001.

**Figure 5:**
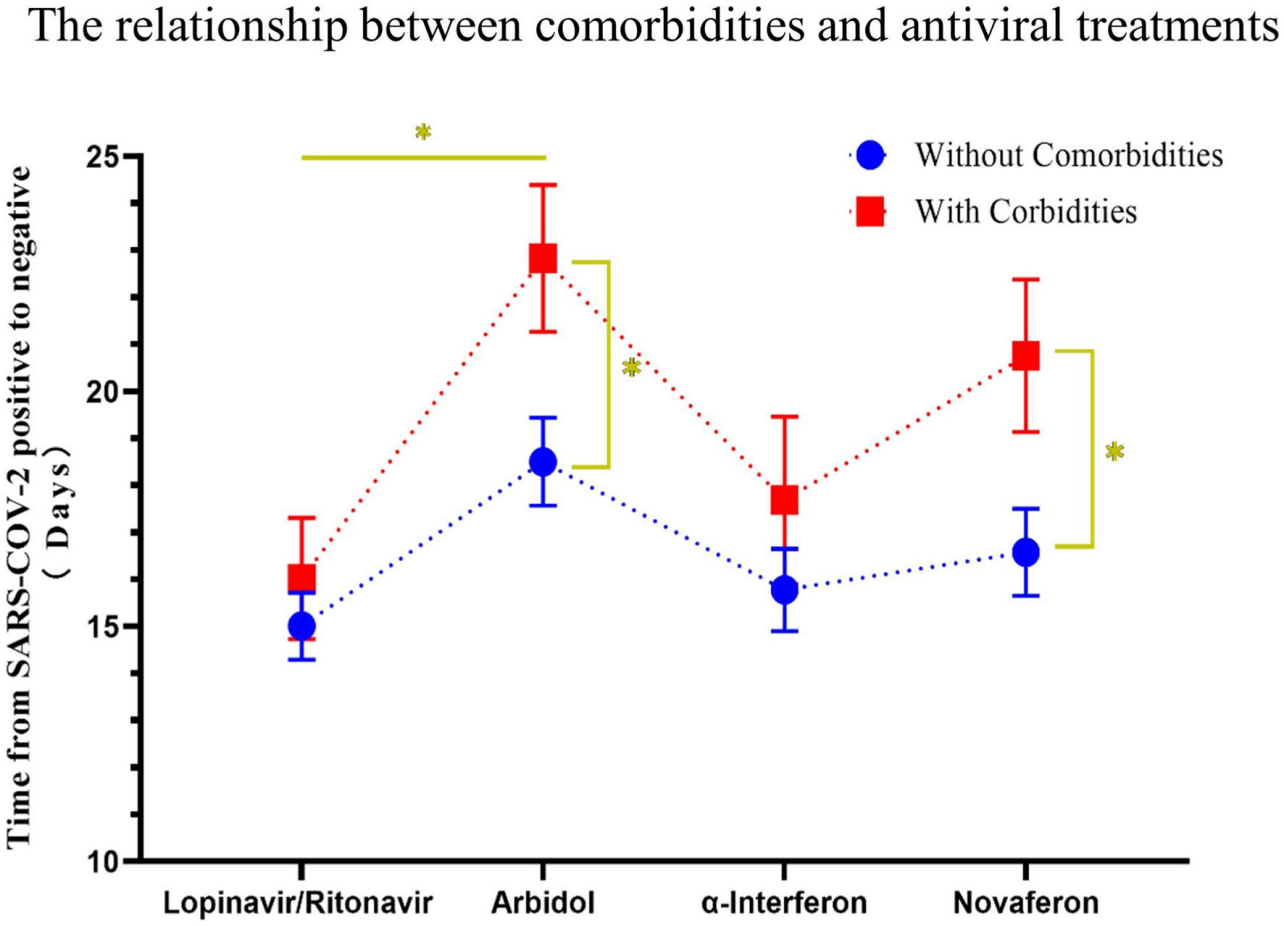
The relationship between comorbidities and the conversion time from real-time PCR (rtPCR) positive to negative during antiviral treatment. Patients were divided by different used antiviral drugs, and the conversion time from rtPCR positive to negative of patients with or without comorbidities was compared. Patients with comorbidities had a longer time of rtPCR conversion than patients without comorbidities throughout antiviral drug treatment. Data are presented as mean ± SEM. The significance was determined as *p < 0.05.

**Figure 6:**
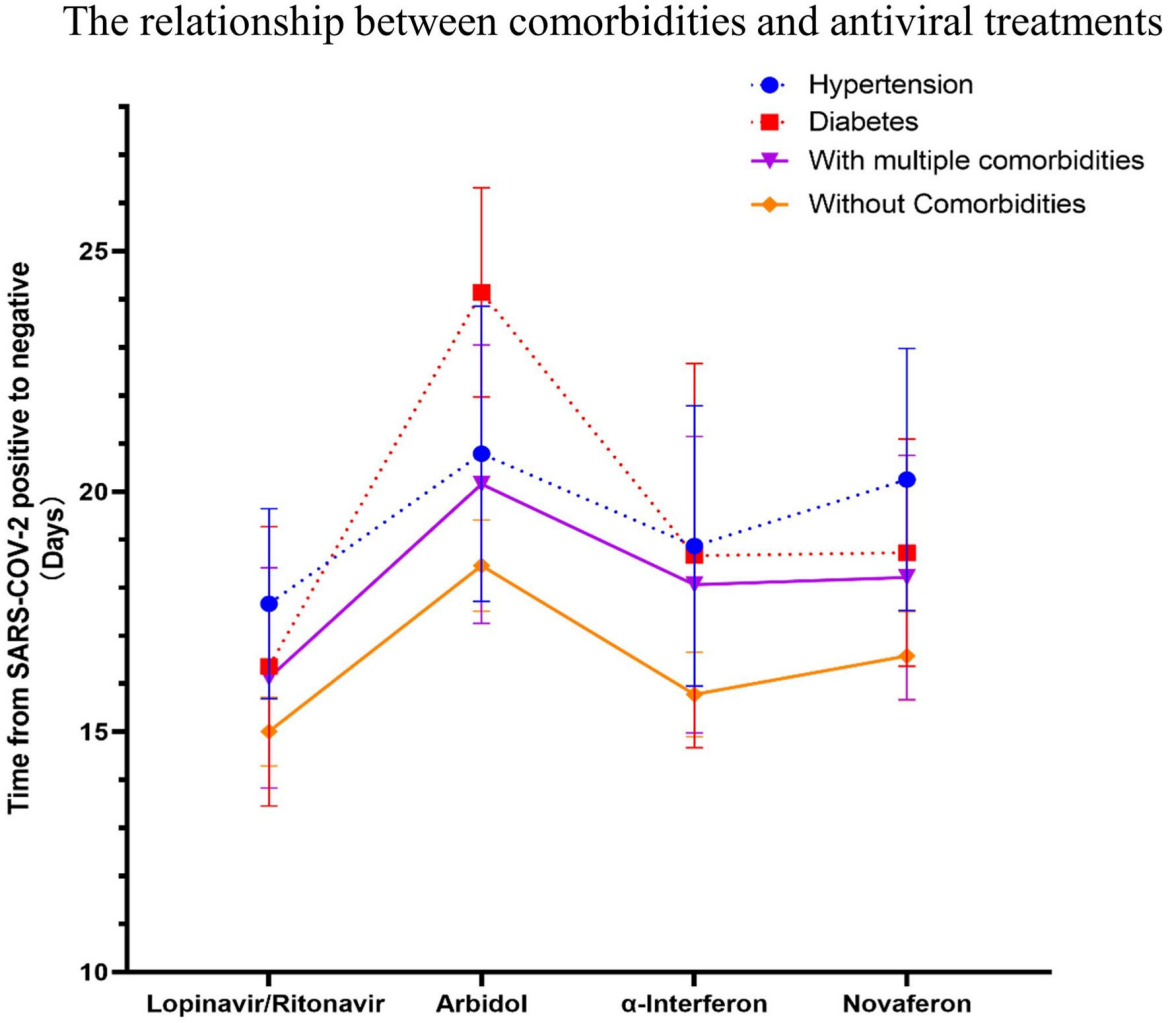
The relationship between specific comorbidities and the conversion time from real-time PRC (rtPCR) positive to negative during antiviral treatment. The time to conversion from rtPCR positive to negative was compared for patients with hypertension, diabetes, multiple comorbidities, and no comorbidities treated with different antiviral drugs. Patients with diabetes had a longer duration of rtPCR conversion than patients without comorbidities in the Arbidol group during antiviral drug treatment (*p* = 0.11). Data are represented the mean ± SEM.

**Figure 7:**
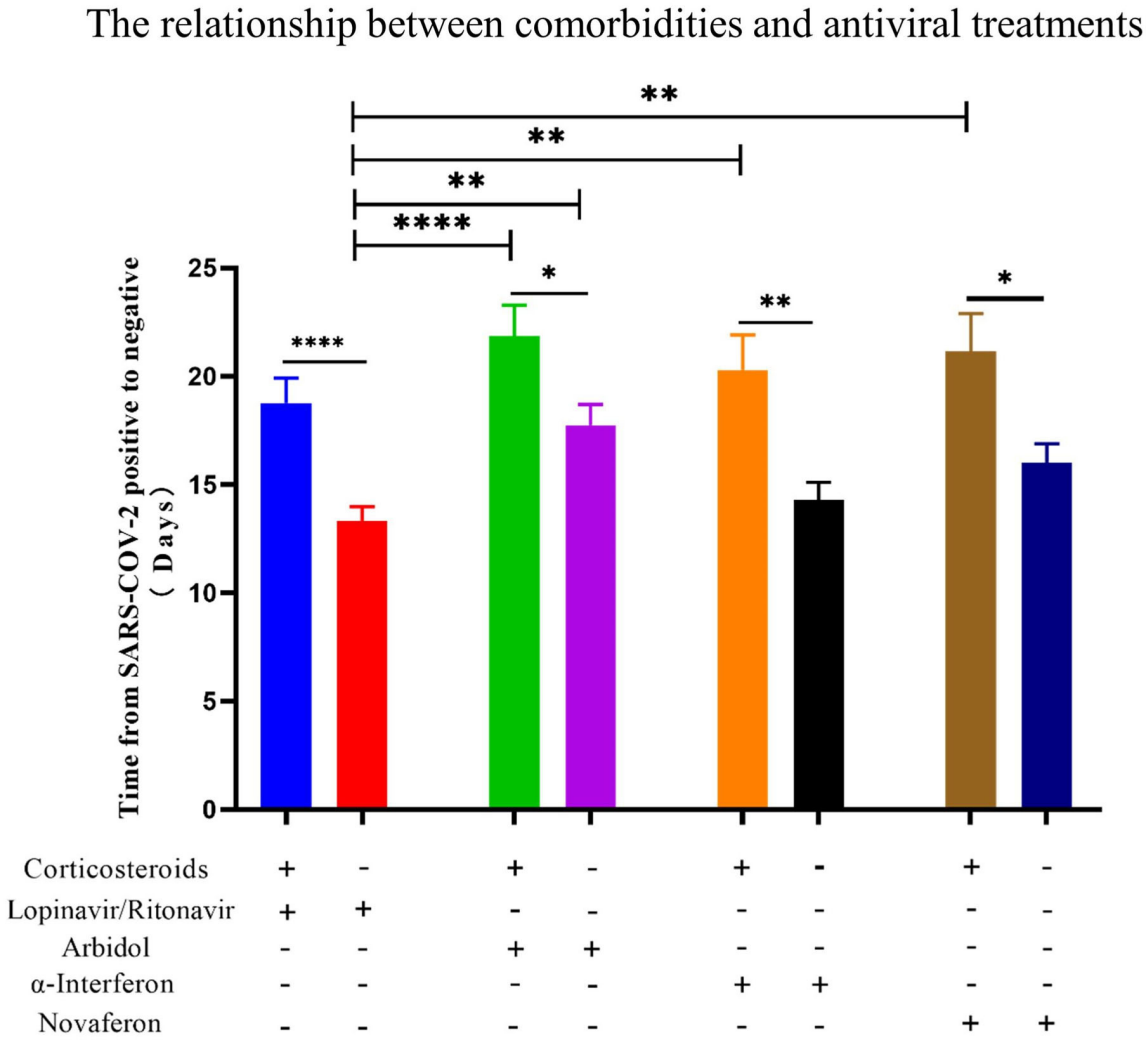
The relationship between the use of corticosteroids and the conversion time from real-time PCR (rtPCR) positive to negative during antiviral treatment. Patients were divided by used antiviral drugs and corticosteroids use. Patients treated with corticosteroids showed in all groups a significantly longer conversion time from rtPCR positive to negative. Data are presented as mean ± SEM. The significance was determined as *p < 0.05, **p < 0.01, and ****p < 0.0001.

There is still an interesting debate as to which treatment strategy would be best for COVID-19 patients. It is now widely accepted that antiviral drugs with or without corticosteroids such as dexamethasone could improve clinical outcome of these patients. Four antiviral drugs (Lopinavir/Ritonavir, α-interferon, Arbidol, and Novaferon) were applied to patients in this study. Every study patient received at least one antiviral drug during hospitalization. Since there was no clear application strategy of antiviral drugs to COVID-19 patients at the beginning of this study, the clinical outcome (symptoms, drug side effects, and nucleic acid PCR result) of the initial given antiviral drug determined further drug administration. We summarized the antiviral drugs (used for patients at least once during hospitalization) applied to patients and their relationship to age, comorbidities, and corticosteroids use to see if that were relevant to the time from rtPCR positive to negative (Table 4). Ninety-four percent of fatalities are uniformly concentrated in the population over 60 years of age; thus, we speculate that age would determine the time of conversion from rtPCR positive to negative. We first analyzed the conversion time using the cutoff age of 30, 50, and 70 and found that patients over 50 years old needed a significantly longer time for SARS-COV-2 conversion (Figure 4A). Therefore, we then compared the duration of rtPRC conversion for patients under and over the age of 50 years among different antiviral strategies (Figure 4B). Comorbidities could be a risk factor for COVID-19 patients (15); therefore, we compared the duration of rtPCR conversion for patients with or without comorbidities (Figure 5). Hypertension and diabetes were the most common comorbidities for patients in our study, so further comparison for patients with hypertension, diabetes, multiple comorbidities, and no comorbidities was made (Figure 6). Moreover, the time of rtPCR conversion was compared for patients with or without corticosteroid used in combination with the antiviral drug (Figure 7). Our data showed that patients over the age of 50, with comorbidities and treated with corticosteroids, needed significantly longer conversion time from rtPCR positive to negative. It is worth mentioning that <10% of patients had diabetes but showed a crucial impact on the duration of rtPCR conversion in the Arbidol group.

**Table 4:** The overview of used antiviral drugs during hospitalization.

**Antiviral drugs**	**Lopinavir/Ritonavir**	**Arbidol**	**α-Interferon**	**Novaferon**
Patients (n)	176	130	117	134
Age (mean)	43.59	45.6	43.7	44.3
Comorbidity (%)	27.8%	26.9%	30.8%	27.6%
Hypertension (%)	13.1%	12.3%	14.5%	11.9%
Diabetes (%)	6.3%	5.4%	5.1%	9.0%
With multiple comorbidities	10.2%	8.4%	12.0%	11.9%
Combination with corticosteroid (%)	37%	35.4%	37.6%	26.1%

## Discussion

SARS-COV-2 is a single-stranded RNA virus (16). Respiratory specimens (pharyngeal swabs, bronchial/alveolar lavage fluid) or blood specimens can be used for rtPCR nucleic acid testing (9). As an objective marker, PCR detection after SARS-COV-2 infection is of great significance for early diagnosis, treatment, and prevention from virus expansion (17). Moreover, the nucleic acid result is a vital reference for recovery if it converses from positive to negative. In the present study, all 239 COVID-19 patients have recovered after treatment, 55.2% of the patients converted from an rtPCR-positive to rtPCR-negative result within 2 weeks, while 44.8% of the patients needed more than 2 weeks.

For a severe virus pandemic situation, medical resources (hospitals, supplies, physicians, nurses, and intensive care unit) are always in high demand. Shortening of the hospital stay could play a crucial role during clinical management of COVID-19; therefore, it is beneficial to determine factors that influence the time from rtPCR-positive to an rtPCR-negative result. As a clinical center for the treatment of COVID-19 in Germany, we have facilitated the cooperative study with a designated hospital for COVID-19 in China in order to analyze treatments carried out at the beginning of the pandemic and learn from them. We believe that this study will provide valuable suggestions for current and future COVID-19 treatments in Europe, China, and the world. In the present study, age, symptoms at onset, inflammation makers, and various treatments of COVID-19 patients during hospitalization were investigated.

The most commonly reported symptoms of COVID-19 are fever, cough, myalgia, and fatigue, whereas less common reported symptoms include headache, diarrhea, hemoptysis, and runny nose (18). Most patients have more than one symptom at admission (10). Similar results were found in our study that fever, cough, and fatigue were the most common symptoms, and the majority of patients experienced at least two symptoms at admission. Moreover, no significant differences were found between the time from rtPCR conversion from positive to negative and the four main symptoms. CT examination is of great importance in the current diagnosis and treatment of COVID-19, which has become one of the most valuable methods for the evaluation of COVID-19 patients (19). Most previous studies demonstrated that COVID-19 patients have typical CT-detectable lung lesions (20, 21), which was also confirmed in our patient cohort and our results (40.2% had single lung lesion and 49.8% had multiple lung lesions). Although the condition of the lung lesions at admission did not affect the timing of rtPCR conversion, patients who progressed lung lesions during hospitalization experienced a significantly longer conversion time than patients without lung lesion progression. This correlation suggests that CT monitoring is important for COVID-19 patients and that lung lesion change is a potential indicator for assessing SARS-COV-2 evolution. Interestingly, two classical inflammation markers (ESR and CRP) in circulating blood were expressed oppositely in COVID-19 patients. Patients with high ESR levels at hospital admission needed more time (>2 weeks) to convert rtPCR result from positive to negative. In comparison, patients with low levels at hospital admission converted from a positive to a negative rtPCR result within 2 weeks. In contrast, CRP showed an opposite tendency, indicating that ESR might be a practical reference indicator for dynamic monitoring of the virus activity in COVID-19 patients. This could be explained by CRP levels falling more quickly than the ESR, normalizing 3–7 days after resolution of tissue injury, whereas ESR can take up weeks to normalize (22). CRP may respond rapidly during the incubation period of SARS-COV-2, while ESR keeps increasing along with the progression of the virus. Further studies are needed to confirm our results and explain the underlying mechanism.

SARS-COV-2 keeps spreading; no designated drug or vaccine has yet been approved to treat human coronaviruses. Lopinavir/Ritonavir, α-interferon, and Arbidol are commonly used to treat SARS-COV-2 infections (23, 24). Novaferon, a novel drug approved for the treatment of chronic hepatitis B in China, exhibits potent antiviral activities and promising antiviral effects on COVID-19 patients (25). A randomized, controlled, open-label trial of 199 hospitalized COVID-19 patients showed no benefit of Lopinavir–Ritonavir to clinical improvement beyond the standard of care, although Lopinavir–Ritonavir was found to have a benefit for some secondary endpoints, and the safety of the treatment was already confirmed (26). α-Interferon and Arbidol are recommended by different research groups to treat COVID-19 (27, 28). All study patients received antiviral drug treatment and converted from SARS-COV-2 positive to negative in our study, confirming the safety of the mentioned antiviral drugs. However, the exact efficacy, combination, and mechanisms of these drugs for SARS-COV-2 still need to be explored. Furthermore, it is noteworthy that patients responded to antiviral drugs differently. We found that patients above 50 years of age, with comorbidities, and the supplementation of corticosteroid needed a significantly longer time to convert from SARS-COV-2-positive to SARS-COV-2-negative result during antiviral treatments.

An analysis of 72,314 diagnosed patients in China showed that people of all ages are generally susceptible. However, the age group 30–79 years accounts for 87%, while children under the age of nine accounts for only 1% (29). Older adults and people with chronic diseases such as asthma, diabetes, and heart disease may be at increased risk of SARS-COV-2 infection (30, 31). Older age has been proven to be a potential risk factor to poor prognosis of COVID-19 patients (31). At the beginning of the epidemic, patients over 70 years with severe symptoms of COVID-19 were classified being at risk and isolated (32). After a few months, the most vulnerable to a COVID-19 infection age group was decreased to 60 years (33, 34). According to the results of our study, the vulnerable age for COVID-19 patients could be even further decreased to 50 years. We found that the rtPCR results in patients over the age of 50 years took longer to convert from positive to negative than in the patient group 50 years and younger. Thus, COVID-19 patients over the age of 50 should practice strict public health measures due to the increased risk of prolonged time to carry the SARS-COV-2 virus and consequently slower conversion from a positive to a negative rtPCR result.

A multitude of previous studies has documented that patients with comorbidities have escalated risks of poorer clinical outcomes with avian influenza, SARS-CoV, and Middle East respiratory syndrome (MERS)-CoV infections. A nationwide analysis of 1,590 COVID-19 patients in China showed that patients with any comorbidity yielded poorer clinical outcomes than those without among laboratory-confirmed cases of COVID-19 (15). A recent meta-analysis found that underlying diseases could be risk factors for severe patients compared with non-severe patients (15). The present study confirms this result, demonstrating that patients with comorbidities needed longer times to convert from a positive PCR result to a negative one. A previous study reported that diabetes could be a significant predictor of morbidity and mortality of COVID-19 (35). Our results showed that diabetic patients treated with Arbidol had a prolonged SARS-CoV-2 conversion, suggesting that the effects of antiviral drugs differ from patients with specific comorbidities. This may be related to the pathological changes in the comorbidities or to the medications for treating comorbidities. One study has shown that the use of antagonists of the renin–angiotensin–aldosterone system in diabetes contributes to a poor prognosis for COVID-19, and treatment of COVID-19 such as antivirals and corticosteroids may worsen glucose control in diabetics (36). Therefore, we suggest that the relationship between the comorbidity treatments and treatments of COVID-19, as well as drug–drug interactions during the treatment of COVID-19, are well worth being further studied.

In the past, corticosteroids were widely used during SARS-CoV1 and the MERS-CoV pandemic (37, 38). A meta-analysis of corticosteroid uses in SARS patients provided conclusive results that may delay viral clearance if given before viral replication is controlled (39). In a retrospective study of 309 critical patients with MERS, nearly half of the patients (49%) were treated with corticosteroids, and these patients were more likely to require mechanical ventilation, vasopressors, and renal replacement therapy (40). In addition, influenza respiratory syncytial virus (RSV) indicated that patients treated with corticosteroids had impaired antibody responses. For COVID-19 patients, corticosteroids are also widely used in septic shock despite uncertainty over their efficacy (41). Dexamethasone is the latest drug touted by experts in the UK as a possible treatment for COVID-19, with evidence suggesting that it can successfully reduce deaths from the virus by up to one-third in critically ill patients (42). According to the latest version of the WHO guidelines for COVID-19, severe and critical COVID-19 patients are recommended to use systemic corticosteroids, while non-severe patients are not recommended to use corticosteroids (43). In the present study, all patients had recovery from COVID-19, and no deaths occurred. Eighty-four percent of patients in our study were non-severed cases. It is shown for recovered patients that it took significantly longer time to reverse SARS-COV-2 from positive to negative in patients who have been treated with corticosteroids than those without during antiviral treatments, indicating that corticosteroids have a delaying effect on the clearance of SARS-COV-2 pathogens. In theory, corticosteroid therapy may bring direct benefit to symptom recovery. However, it may act as an immunosuppressive agent and not only inhibits inflammation of the lung but also suppress the immune response and pathogen clearance, therefore delaying the recovery. Overall, corticosteroids are proven to reduce the mortality of inpatients with critical COVID-19; however, corticosteroids use is likely to prolong the duration from SARS-COV-2 positive to negative, especially for non-severe COVID-19 patients. Therefore, corticosteroids should be used with caution for non-severe COVID-19 patients.

## Conclusion

rtPCR test is of great importance to evaluate COVID-19 patients, serving as a reliable indicator of SARS-COV-2 clearance in patients. It is of great benefit to reduce the time of SARS-COV-2 clearance and thus shorten the hospital stay of COVID-19 patients to save medical resources. ESR could be an indirect indicator to monitor SARS-COV-2 activity in patients over time. During antiviral treatment, COVID-19 patients at the age of over 50, having comorbidities, and/or being exposed to a corticosteroid due to respiratory dysfunction will most likely have a prolonged conversion from rtPCR positive to negative.

## Data Availability Statement

The raw data supporting the conclusions of this article will be made available by the authors, without undue reservation.

## Ethics Statement

The studies involving human participants were reviewed and approved by The First Hospital of Changsha Hospital Ethics Committee. Written informed consent to participate in this study was provided by the participants' legal guardian/next of kin.

## Author Contributions

SZ: writing—original draft and review and editing. YH: writing—original draft and data curation. WT: data curation. AN: writing—review and editing, and conceptualization. FZ: data curation and conceptualization. All authors contributed to the article and approved the submitted version.

## Conflict of Interest

The authors declare that the research was conducted in the absence of any commercial or financial relationships that could be construed as a potential conflict of interest.
